# Severe atherosclerosis of donor hepatic arteries is a salvageable condition in liver transplantation to optimise the graft utilisation: A case series and review of the literature

**DOI:** 10.1016/j.ijscr.2019.05.048

**Published:** 2019-05-31

**Authors:** Yuhki Sakuraoka, Amanda Pinter Carvalheiro da Silva Boteon, Rachel Brown, M. Thamara P.R. Perera

**Affiliations:** a Second Department of Surgery, Dokkyo Medical University, Japan; b The Liver Unit, University Hospitals Birmingham NHS Foundation Trust, Queen Elizabeth Hospital Birmingham, United Kingdom; c Department of Pathology, University Hospitals Birmingham NHS Foundation Trust, Queen Elizabeth Hospital Birmingham, United Kingdom

**Keywords:** LT, liver transplantation, ECD, extended criteria donors, MLs, marginal livers, PBC, primary biliary cirrhosis, PHT, portal hypertension, UKELD, United Kingdom Model for End-Stage Liver Disease, AST, aspartate aminotransferase, ALT, alanine aminotransferase, ALP, alkaline phosphatase, INR, international normalized ratio, BMI, body mass index, DBD, donation after brain stem death, DCD, donation after circulatory death, GDA, gastroduodenal artery, CHA, common hepatic artery, CIT, cold ischaemia time, WIT, warm ischemia time, HAT, hepatic artery thrombosis, Endarterectomy, Liver transplantation, Atherosclerosis, Case report

## Abstract

**Introduction:**

The presence of atherosclerosis of the common hepatic artery (CHA) in donor livers potentially contributes to vascular complications after liver transplantation, thereby most of those organs are traditionally discarded. Herein, we describe the successful outcome of three patients transplanted with grafts that had severe atherosclerosis of the donor CHA up to the level of the gastroduodenal artery (GDA).

**Presentation of case:**

In all three cases, endarterectomies were performed by dissection between the atheromatous core and the artery intima using a dissecting spatula, allowing to secure the lumen of the vessel. The native CHA/GDA patch was aligned with the corresponding CHA/GDA patch from the graft for the arterial reconstruction. No vascular complications were seen post-operatively.

**Discussion:**

Endarterectomy and anatomical reconstitution of the arterial tree, without any redundancy or kinking, allowed for the successful transplantation of organs that would be otherwise discarded. Further, the straight alignment of the arteries may enhance flow dynamics, preventing thrombosis.

**Conclusion:**

This report might guide future studies targeting means to increase the utility of donor livers discarded due to arterial atherosclerosis.

## Highlights

• We showed how to manage sever atherosclerosis in liver transplant with special technique.
• This case report will help not to discard donated liver graft.
• Management of sever artherosclerosis, liver transplantation.

## 1 Background

The current demand for donor organs in liver transplantation (LT) largely exceeds the supply [1]. In the United Kingdom (UK) between 2016 and 2017, around 15% of patients died or were removed from the waiting list because they became too sick [2]. Therefore, transplant centres are increasingly accepting livers from extended criteria donors (ECD), including old donors and donation after circulatory death (DCD) [3,4]. In the current era, the number of patients suffering from lifestyle-related co-morbidities such as obesity, hypertension and diabetes mellitus is rising, and they are already major causes of death in some countries [[5], [6], [7]]. These diseases are usually associated with atherosclerosis of vessels, which would add more risks in terms of graft quality when considering those individuals as potential organ donors.

Atherosclerosis’ impacts graft outcomes after LT in many ways. Usually, atherosclerosis is confined to the ostia of origin of visceral vasculature, such as the celiac axis or renal arteries, and it may cause poor organ perfusion during procurement. Extensive atherosclerosis of the common hepatic artery (CHA) can lead to vascular complications after LT, including hepatic artery thrombosis (HAT), which ranges from 2% to 9% [[8], [9], [10]]. Progression of donor atherosclerosis and neo-intimal hyperplasia causing hepatic artery stenosis after LT are major potential complications that may result in ischemic biliary complications and long-term graft loss [11,12]. Therefore, severe atherosclerosis of the CHA is considered a reason for organ discard due to the potential hazards described above.

The arterial reconstruction during graft implantation depends on a combination of factors, including donor and recipient artery diameters, vessel quality, donor/recipient anatomical variations and specific conditions related to the indication for LT [13,14]. In this report, we describe three cases of grafts that had severe atherosclerosis of the CHA as distal as the origin of the gastroduodenal artery (GDA). Endarterectomy, followed by arterial reconstruction, was performed, allowing for a successful outcome in transplanted patients. This was possible due to the relatively unconventional, but anatomically favourable, arterial reconstruction techniques that were performed. The work has been reported in line with the PROCESS criteria [15].

The early success and technical aspects are described herein and enlighten the readership on careful consideration of such grafts for clinical transplantation.

## 2 Case presentation

### 2.1 Case 1

The patient was a 51-year-old, female sex, blood group O, with advanced decompensated primary biliary cirrhosis presenting with refractory ascites, sarcopenia, portal hypertension and significant jaundice. The United Kingdom Model for End-Stage Liver Disease (UKELD) score was 61 [[16], [17], [18]] and she was listed for LT. This patient received a DCD liver from a 74-year-old, male sex, donor. Further donor details are presented in Table 1. Recipient laboratorial data on the index admission for transplantation were summarised in Table 2.

**Table 1:** Donor demographic data.

Characteristics	Case 1	Case 2	Case 3
Donor Type	DCD	DBD	DBD
Cause of Death	Intracranial thrombosis	Intracranial haemorrhage	Intracranial thrombosis
Age (years)	74	48	52
Sex	Male	Female	Male
Blood Group			
ABO	O	B	O
Rhesus	+	+	+
BMI (kg/m^2^)	22	23	31
Comorbidities			
*Hypertension*	+	+	+
*Hypercholeterolemia*	+	+	+
*Type II diabetes*	−	+	−
*Smoking*	+	+	+
*CKD*	−	+	−
*Heart disease*	−	−	+
Donor WIT^a^	17	--	--

**Abbreviations: DCD**: donation after circulatory death; **DBD**: donation after brain stem death; **CKD**: chronic kidney disease; **WIT**: warm ischemia time.

a Donor warm ischaemic time was defined as the interval between the systolic blood pressure less than 50 mmHg or/and arterial oxygen saturation to less than 70% to commencing the aortic cold perfusion in the donor.

**Table 2:** Operative timings and post-operative biochemistry data.

Operative timings			
	Case 1	Case 2	Case 3
CIT (min)	403	595	630
Implantation time^b^ (min)	21	31	25
Operative time (min)	247	317	332

Post-operative biochemistry data									
	Case 1			Case 2			Case 3		
*Parameter*	*Adm*	*1m*	*3m*	*Adm*	*1m*	*3m*	*Adm*	*1m*	*3m*
**AST** (IU/L)	128	–	30	–	34	43	43	39	17
**ALT** (IU/L)	90	71	20	2.,965	14	19	23	47	16
**Bil** (mmol/L)	207	240	18	68	29	12	67	17	8
**ALP** (IU/L)	338	1,585	429	86	1044	329	136	183	82
**INR**	1.5	1.2	1.0	4.5	1.1	1.0	1.7	2.2^a^	2.2^a^

**Abbreviations –** CIT: cold ischemia time, which is defined as the time that an organ surgically removed for transplantation remains in a chilled perfusion solution before engraftment, Adm: admission, AST: enzymes aspartate aminotransaminasetransferase, ALT: alanine aminotransferasetransaminase, γ-GTP: γ-glutamyl transpeptidase, Bil: total bilirubin, ALP: alkaline phosphatase, PT-INR: prothrombin time International normalized ratio.

a The patient was treated with warfarin.

b Until portal vein reperfusion.

The graft had normal hepatic artery anatomy, but an extensive atheromatous plaque up to the GDA. During implantation, the graft GDA was divided obliquely along the main hepatic artery stem and an endarterectomy was done. A plaque free portion of the hepatic artery above the GDA was obtained for direct anastomosis to the native CHA at the GDA junction; thereafter, a short, straight and non-redundant arterial reconstruction was performed. The anastomosis width was just over 6 mm, there were good pulse waves and the resistance index was confirmed by doppler ultrasound intra-operatively.

**Fig. 1:**
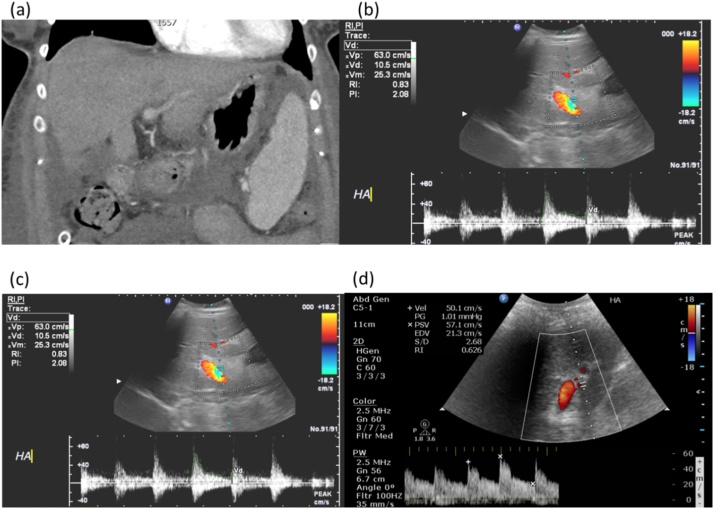
Post-operative imaging exams. Computerized tomography at post-operative day 11 showed the site of arterial anastomosis (white arrow) naturally aligned without redundancy (*panel a*). Ultrasonography performed for case1 (*panel b*), case 2 (*panel c*) and case 3 (*panel d*) at post-operative day 11 showed resistance index of 0.83, 0.54 and 0.63, respectively. For all cases the arterial flow was normal without evidence of hepatic artery thrombosis.

**Fig. 2:**
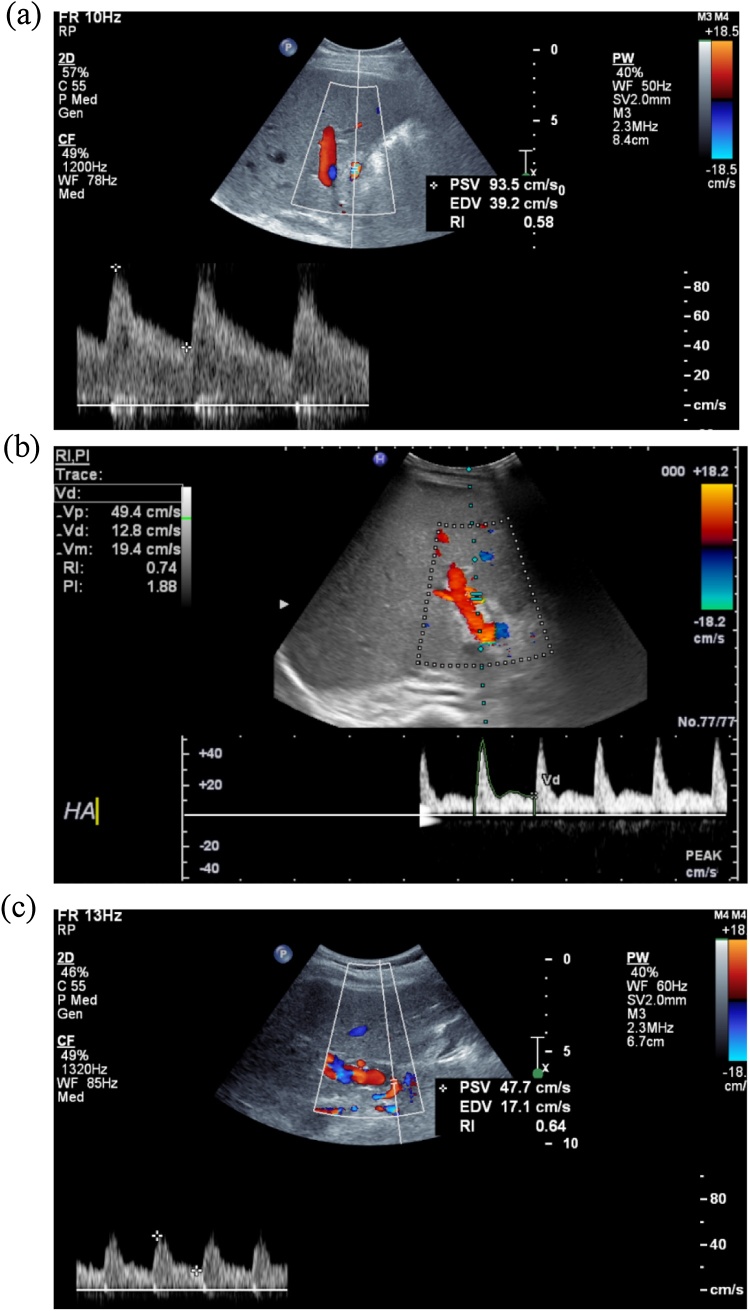
Colour-doppler-ultrasonographic images at late post-operative course; for case 1 (*panel a*) at post-operative day (POD) 38, case 2 (*panel b*) at POD55 and case 3 (*panel c*) at POD90. All scans showed adequate flows on the hepatic artery with resistance index within normal range.

Surgical times are presented in Table 2. In terms of postoperative complications, this patient developed renal dysfunction and fluid overload in the immediate post-operative period, requiring temporary renal support. Additionally, further respiratory infection required the intensive care unit (ICU) up to post-operative day (POD) 9. Patient also developed delayed graft function with prolonged cholestasis. The bilirubin level was 266 mmol/L on POD 29 and it improved gradually down to 69 mmol/L by POD 49 when the patient was discharged. It was within the normal range (18 mmol/L) after 3 months of the transplant. During hospitalisation 4500 units of low molecular heparin and 75 mg of aspirin were given daily from POD 1 onward, as per unit protocol. The haemoglobin level was maintained around 80 g/L and platelets on an average of 80,000 counts until POD 5. Immunosuppression was standard, and the biochemistry was within the normal range at 4 months follow up. An ultrasound scan at 4 months post-transplant showed patent graft vessels and a non-dilated biliary system without evidence of HAT or hepatic artery stenosis (Fig. 1, Fig. 2).

### 2.2 Case 2

A 20-year-old patient presented with acute liver failure of uncertain aetiology associated with acute kidney injury due to poor oral intake and vomiting. She was admitted to the ICU with alanine aminotransferase (ALT) levels of 4000 IU/L, international normalized ratio (INR) of 6.0 (Table 2), mental confusion and hypoglycaemia. A comprehensive liver autoimmune and viral serology was pending, and the toxic screen was negative. Patient was managed with hemofiltration, received *N*-acetylcysteine with antimicrobial cover and listed for super-urgent liver transplantation. A liver from a donor after brain stem death (DBD), 48-year-old, female sex, was offered for this patient (donor details in Table 1).

The graft hepatic artery had atheromatous plaque beyond the GDA in the proper hepatic artery (PHA). Graft hepatic artery was prepared at the GDA, and endarterectomy was performed. Recipient CHA was prepared at the GDA junction and arterial reconstruction was done with an anastomosis just over 5 mm wide. The plaque removed from the donor CHA/GDA junction had 1.2 × 0.3 × 0.2 cm and it was reported as an organised atheromatous plaque on histology. The remnants of the donor artery discarded and sent for histology showed extensive atheromatous changes with intimal thickening and infiltration of foam cells and cholesterol clefts. Focally, in one of the branches, there was at least 75% luminal occlusion. Surgery timings are presented in Table 2.

After the liver transplant, the patient required inotropic and ventilation support for 2 days, with subsequent uneventful postoperative course. She was discharged on POD 14. The same protocol of thrombosis prophylaxis previously reported has been followed.

**Fig. 3:**
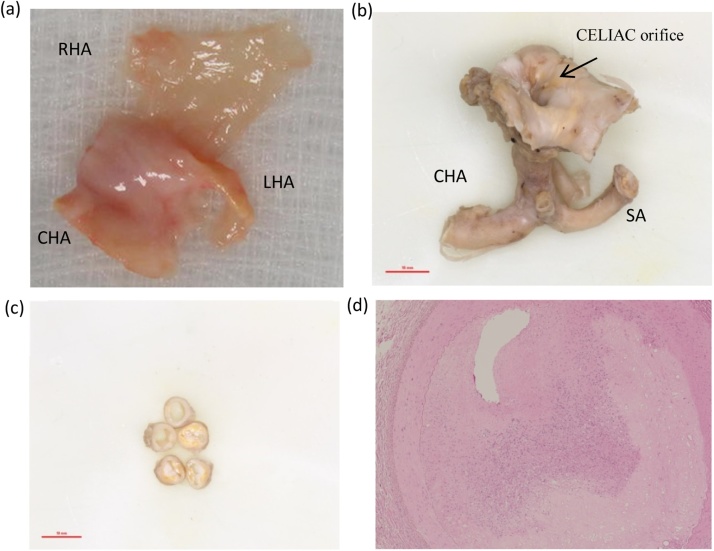
Macroscopic and histologic assessment of atherosclerotic donor liver vessels. panel (a) shows the plaque removed in block from case 3, extending towards the left, right hepatic artery (LHA and RHA, respectively) from the common hepatic artery (CHA) main stem. The atheromasclerotic lesion from case 2 is represented in panel (b), it started at the origin of the celiac trunk extending to just beyond the bifurcation of the CHA and splenic artery (SA). Panel (c) shows cross sections of the arteries from case 2 with macroscopic severe occlusive lesion thickening the vessel’s wall. Histological assessment (Panel d) found that the lesion was occluding 75% of the lumen of the artery.

Biochemical values were within normal range after 3 months of the transplant (Table 2). An ultrasound scan showed patent graft vessels and a non-dilated biliary system without evidence of HAT (Fig. 1, Fig. 2). The patient is well after 3 months follow up (Fig. 3).

### 2.3 Case 3

A 56-year-old patient, blood group O, diagnosed with alcohol-related liver disease, presenting with a UKELD of 49 that had previously undergone laparotomy for diverticular abscess and reversal of Hartmann’s procedure, was listed for LT. He received a DBD liver from a 74-year-old, male sex donor, who died for intracranial thrombosis (donor details in Table 1).

The graft arterial tree was completely atheromatous from the aortic patch, way up to the PHA, beyond the GDA, and into the left hepatic artery (LHA) and right hepatic artery (RHA) branches. This was not informed by the retrieval or bench picking teams, and therefore, was recognized only during implantation once the surgeon was ready for the arterial reconstruction following portal reperfusion. The lumen of the atheromatous proper hepatic artery was significantly narrow, and the disease was circumferential. Surgical endarterectomy of the PHA was performed and the plaques extending up in both branches were removed en-mass. The total length of the endarterectomized specimen was 1.5 cm upwards from the GDA of the graft. Distal hepatic artery, following the procedure, was soft. The native CHA was prepared at the GDA junction. Further, the hepatic arteries were aligned straight, and they were reconstructed with 7-0 prolene with a continuous suture. The intra-operative doppler ultrasound showed good flows on the right, left and segment 4 artery branches. Histological assessment reported extensive atheromatous change, markedly thickened intima with myxoid change and foam cells. Occlusion the lumen of 50% was found in some portions.

This patient had an uneventful post-operative course, stayed in the ICU for one day and was discharged at POD 8. He was at a higher risk of artery thrombosis because the atheromatous plaques were extending up to the LHA and RHA branches, hence short-term anticoagulation with warfarin was undertaken post-operatively. The targeted INR was between 2.0 and 2.5 over the three initial months after transplantation. Biochemistry values were within a normal range at 3 months post-transplant (Table 2), and an ultrasound scan showed patent graft vessels and no evidence of HAT (Fig. 1, Fig. 2). The patient is well at a follow-up of three months.

## 3 Discussion

Atherosclerosis is a disease that occurs in elastic arteries, such as the aorta, or in medium-sized muscular arteries, and elevated lesions of the tunica intima called plaques characterize it. Morphologically, it is classified into four stages: fatty streaks, fibrous plaques, atheromatous plaques and complicated lesions [19]. Atherosclerotic plaques develop when endothelial cells are injured by haemodynamic or other kinds of stress. Plaque gradually increases through lipid deposition and an increased extracellular matrix, in addition to migrating macrophages and smooth muscle cells. If an erosion or an ulcer develops on the surface of a plaque, a blood clot forms, and organization and calcification can occur. When this process is repeated, it results in complicated lesions [20]. Accordingly, plaque removal during arterial anastomosis is essential to prevent postoperative thromboembolism.

**Fig. 4:**
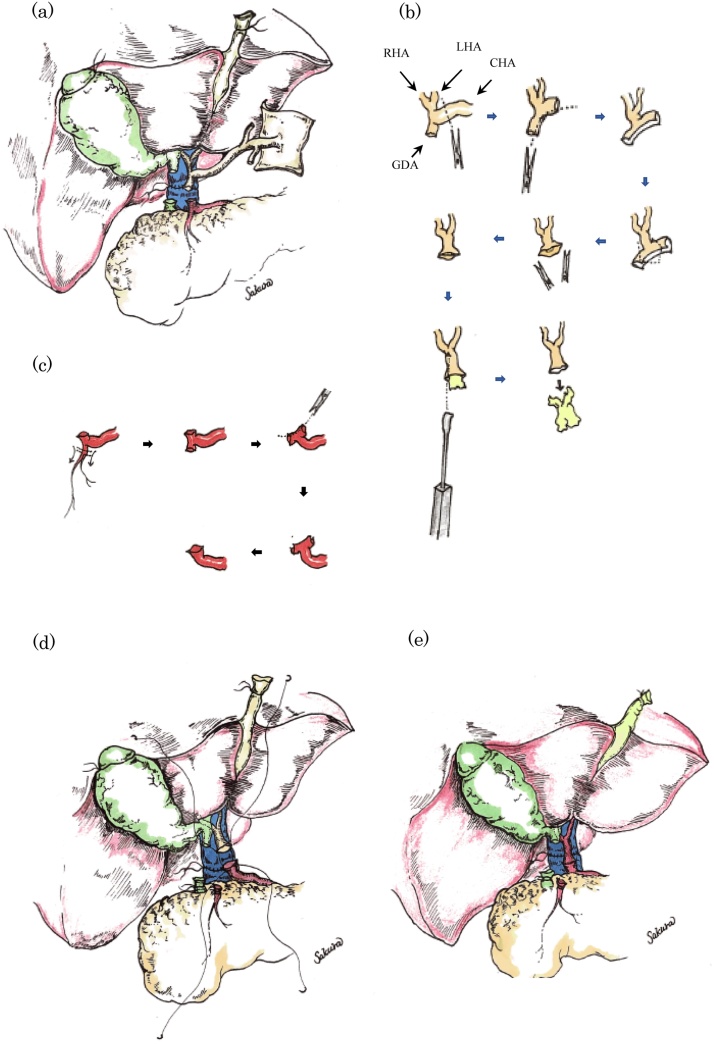
Schematic representation of the surgical technique for management of atherosclerotic arteries in liver transplantation. Once the liver graft was implanted and reperfused, the site for arterial anastomosis was then decided (panel a). An oblique cut at the bifurcation of the gastroduodenal artery (GDA)/common hepatic artery (CHA) was made for creation of a wide patch. Endarterectomy was performed by dissection between the atheromatous core and the artery intima using a dissecting spatula, allowing to secure the lumen of the anastomotic vessel (panel b). Recipient GDA was cut and mobilised to make a patch at the native side (panel c). Subsequently, end-to-end anastomosis was performed on a continuous fashion (panel d) with no kinking and natural flow (panel e).

In the three cases reported, the arteries chosen for anastomosis had severe atherosclerosis and, technically, unique steps were followed. In all three cases, the native CHA was prepared at the GDA level after dividing from the distal GDA and transfixing it. A wide arterial patch was created by opening the GDA patch. The key rationale was to align this native CHA/GDA with the corresponding CHA/GDA patch from the graft. This is a natural anatomical reconstitution of the arterial tree without any redundancy or kinking, which straight aligns the arteries and thus, enhances flow dynamics, preventing thrombosis. The GDA/CHA patch was created, and a wide Carrel patch was obtained from the grafts before endarterectomy. Endarterectomies were performed by dissection between the atheromatous core and the artery intima using a dissecting spatula, allowing to secure the lumen of the vessel. Preservation of the tunica media, including the elastic fibres, is crucial for maintaining the elasticity of the arteries. Residual plaque is a risk for postoperative thrombosis and thus, its total removal is considered essential (Fig. 4).

Endarterectomy was first carried out on a superficial femoral artery in 1946 by Cid dos Santos and subsequently, on the abdominal aorta by Wylie in 1951 [21,22]. During the 1950s and 1960s, aortoiliac endarterectomy (AIE) was the standard procedure for treatment of aortoiliac occlusive disease [[23], [24], [25]]. Connolly JE et al. (2006) reported a rate of 89.2% patency of aorto-common iliac endarterectomy aortoiliac occlusive disease [26]. In 2014, Siracuse JJ et al. investigated a total of 1513 patients and found that common femoral endarterectomy is well tolerated by the majority of patients with peripheral arterial disease [27]. As for a transplant, Yuan XP et al. (2012) revealed an adequate graft survival rate in 62 diabetic recipients with endarterectomy for iliac atherosclerosis before renal arterial anastomoses [28]. Despite endarterectomy being an established procedure, it is not reported in LT, and we described for the first time the use of endarterectomy for atherosclerosis of donor liver.

Many transplant surgeons have chosen the splenic artery patch or the CHA stem from the graft for arterial reconstruction, whereas we opted for the GDA patches. We believe that avoiding kinking and aligning the arteries contributes to the long term patency of graft vessels. Flow and pulse wave are routinely checked by intra-operative and post-operative ultra sound examinations (Fig. 1, Fig. 2). These are also fundamentally important to assess whether optimal arterial reconstruction was and to rule out early HAT. The contentious use of short to medium term anti-coagulation was based on the risk balance. Anti-coagulation treatment has its own risks, hence we consider this on a case by case basis.

## 4 Conclusion

In conclusion, we reported three cases of severe atherosclerosis of donor hepatic arteries recognised during LT, where pragmatic and judicious decision making enabled successful endarterectomy and appropriate arterial reconstruction. All three grafts were under different circumstances, from urgent to elective transplantation, yielding successful transplantation through technical modification, demonstrating potential avenues to increase the utility of otherwise discarded donor livers.

## Conflicts of interest

I have no financial relationships to disclose and there are not any financial and personal relationships with other people or organisations about all authors.

## Funding

I have no financial relationships to disclose and there is not any sponsor with funding.

## Ethical approval

Our reported case here involves a sufficient ethnical level. The patient was approved to be on the published paper through our formed document at our institute. The patient showed their willingness to be reported by medical paper or presentation via our document approved by our institute.

In addition, we obtained ethical certification with the approval number CARMS-14495 from ethic committee in University Hospitals Birmingham NHS Foundation Trust, Queen Elizabeth Hospital Birmingham, United Kingdom.

## Consent

Fully informed consent was obtained with some document. Written informed consent was obtained from all patients for publication of this case series and accompanying images. We confirm that have written consent for all patients A copy of the written consent of all patients is available for review by the Editor-in-Chief of this journal on request. Moreover, we obtained ethical certification with the approval number CARMS-14495 from ethic committee in University Hospitals Birmingham NHS Foundation Trust, Queen Elizabeth Hospital Birmingham, United Kingdom.

## Author contribution

Yuhki Sakuraoka: Literature review and writing the article.

Amanda Pinter Carvalheiro da Silva Boteon: mainly managed the patient and led the patient to discharge earlier as well as they helped author collect the clinical data smoothly.

Rachel Brown: the pathologist detected the specific pathological features and provided all microscopic pictures.

M Thamara PR Perera: Editing the article mainly and he per-formed the surgery. He is the chief consultant surgeon of liver transplant.

## Registration of research studies

Our reported case here is not research study. This is a case series.

Moreover, we obtained ethical certification with the approval number CARMS-14495 from ethic committee in University Hospitals Birmingham NHS Foundation Trust, Queen Elizabeth Hospital Birmingham, United Kingdom.

In addition to this, we participated in Research Registry with the approval number “researchregistry4629” to be publicly accessible.

## Guarantor

Dr Yuhki Sakuraoka is the Guarantor of this report and has full responsibility to it.

## Provenance and peer review

Not commissioned, externally peer-reviewed.
